# Dr. W. Edson Apple

**Published:** 1914-04

**Authors:** 


Dr. W. Edson Apple, Jefferson, 1898, of Philadelphia, died at the Episcopal Hospital of that city, February 15, aged 36. He was formerly a surgeon in the U. S. Army and was well known to Buffalo physicians through that connection.

